# Keystone Veterinary Medical Association

**Published:** 1901-12

**Authors:** E. M. Ranck

**Affiliations:** Secretary


### KEYSTONE VETERINARY MEDICAL ASSOCIATION.

The regular monthly meeting of this Association was held on November 12, 1901, at the northwest corner of Broad and Filbert Streets, Philadelphia, Pa., with the following members of the profession in attendance: Drs. Bunting, Carter, Drake, Fuller, Flood, Gavin, Gorman, Kooker, Lintz, Marshall, Hoskins, Pearson, Williams, Rhoads, Ridge, and Ranck; also about fifteen students of the University of Pennsylvania Veterinary Department. The minutes of the previous meeting were read and adopted. Communications read and acted upon separately, one of which was from one of the oldest and most active members, Dr. James B. Rayner, of West Chester, who wished to resign owing to impaired health. A motion to accept his resignation was carried, and a motion to elect him to honorary membership was also carried. The Rules and By-laws having been suspended, he was unanimously elected an honorary member.

A very interesting paper was read by Dr. Bunting, of New Jersey, in which he cited numerous cases of failure from the tuberculin test, also a peculiar affection of the heart, which he attributed to the tuberculin This paper elicited a great amount of discussion, in which all the members participated, and was finally closed by Drs. Pearson and Hoskins. A vote of thanks was extended Dr. Bunting by the Society for his interesting paper.

The other essayists being unavoidably absent, the Chair asked for reports of cases in which the topic of "The Most Humane and Efficient Methods of Killing Animals" was propounded by Dr. Williams. This again brought forth many different methods, but the majority present seemed to favor "chloroform" for small animals, while for cattle and horses "strychnine sul." in sufficient doses, intravenously, seemed to be the best method. This topic is one of great interest, and should be discussed by other veterinary associations, so the profession throughout the country could be properly instructed.

Dr. Kooker reported an interesting case of purpura hemorrhagica, which developed marked symptoms of azoturia, and, strange to say, is getting better, and a complete recovery seems certain.

Dr. Hoskins, who was the Pennsylvania representative on the Committee of Arrangements for our last annual meeting of the A. V. M. A., reported he has a balance of $30 after all expenses had been paid, and asked what the members suggested. He stated that a certain member of the profession was in need of money, and being an invalid had no earning capacity, and, furthermore, this member had been for many years a very earnest worker and active practitioner, so he suggested we donate to him one-half of the above sum.

The Secretary suggested that probably the next in turn to be a beneficiary, who was also in urgent need of funds, was the Keystone Veterinary

Medical Association, and upon the suggestion a motion was made and carried that the remaining $15 be donated to this Association.

Dr. Pearson offered the following resolution:

*Resolved*, That the President of the Keystone Veterinary Medical Association is hereby authorized and requested to appoint a special committee of five to investigate and report upon matters pertaining to the milk supply of Philadelphia, as follows:

1. To what extent are the claims of Philadelphia milk dealers in respect to the sanitary and veterinary inspection of their dairies justified?

2. What precautions are used by managers of hospitals to procure pure and wholesome milk for the use of the persons under their care?

Interspersed with the above business the Entertainment Committee, under the leadership of our President, Dr. Rhoads, furnished us with a delightful luncheon, with the necessary addenda to make the meeting something more than a dry, dull, business affair.

The Secretary announced the programme as far as could be determined at this time, after which we adjourned at 11 P.M.

E. M. RANCK,
Secretary.

---

